# Management of IgG4-related cholangitis: diagnosis, therapy, and long-term surveillance

**DOI:** 10.1093/gastro/goaf032

**Published:** 2025-04-04

**Authors:** Toni Herta, Maik Schröder, Dominik Geisel, Cornelius Engelmann, Frank Tacke

**Affiliations:** Department of Hepatology and Gastroenterology, Charité–Universitätsmedizin Berlin, Campus Virchow-Klinikum (CVK) and Campus Charité Mitte (CCM), Berlin, Germany; Berlin Institute of Health at Charité–Universitätsmedizin Berlin, BIH Biomedical Innovation Academy, BIH Charité Clinician Scientist Program, Berlin, Germany; Department of Hepatology and Gastroenterology, Charité–Universitätsmedizin Berlin, Campus Virchow-Klinikum (CVK) and Campus Charité Mitte (CCM), Berlin, Germany; Department of Radiology, Charité–Universitätsmedizin Berlin, Berlin, Germany; Department of Hepatology and Gastroenterology, Charité–Universitätsmedizin Berlin, Campus Virchow-Klinikum (CVK) and Campus Charité Mitte (CCM), Berlin, Germany; Department of Hepatology and Gastroenterology, Charité–Universitätsmedizin Berlin, Campus Virchow-Klinikum (CVK) and Campus Charité Mitte (CCM), Berlin, Germany

**Keywords:** IgG4-related cholangitis, IgG4-RD, cholangiocarcinoma, primary sclerosing cholangitis, pancreatic ductal adenocarcinoma, immune suppression

## Abstract

IgG4-related cholangitis (IRC) is a chronic cholestatic liver disease that often occurs concomitantly with autoimmune pancreatitis type 1. Both conditions are manifestations of IgG4-related disease, a systemic autoimmune-mediated fibroinflammatory disorder. Patients often present with jaundice and weight loss, mimicking hepatobiliary malignancies, such as cholangiocarcinoma, primary sclerosing cholangitis, and pancreatic cancer. Accurate diagnosis is challenging due to the absence of pathognomonic findings but can be achieved using the HISORt criteria (histology, imaging, serology, other organ involvement, and response to immunosuppressive therapy). Early diagnosis is critical to avoid unnecessary surgery and prevent progression to liver fibrosis or cirrhosis. IRC responds well to corticosteroid therapy, though relapses are common, necessitating long-term immunosuppressive treatment in many cases. Steroid-sparing agents for remission induction and maintenance therapy comprise immunomodulators, such as azathioprine, as well as B-cell depletion therapies, such as rituximab. This review provides a structured clinical overview of the diagnosis, differential diagnosis, and therapy, including novel therapeutic options, such as inebilizumab, for this rare yet severe condition. A key focus is on long-term surveillance strategies, which include laboratory tests, imaging (contrast-enhanced magnetic resonance imaging/magnetic resonance cholangiopancreatography, ultrasound, endosonography), and, particularly in patients with fibrotic bile duct strictures, endoscopy (endoscopic retrograde cholangiopancreatography, cholangioscopy).

## Introduction 

IgG4-related cholangitis (IRC) is an autoimmune-mediated inflammatory disease of the bile ducts, characterized by typical histopathological changes and often, but not necessarily, elevated IgG4 serum levels [1–3]. IRC is the hepatobiliary manifestation of IgG4-related disease (IgG4-RD), a systemic inflammatory and fibrosing disorder of unknown cause. Clinically, IgG4-RD presents in a highly variable manner due to its potential to involve multiple organs [1]. The vast majority of patients (>90%) with IRC simultaneously exhibit clinical, imaging, or histological changes consistent with IgG4-related autoimmune pancreatitis (AIP) type 1, the most common manifestation of IgG4-RD in the gastrointestinal tract [4]. IRC can manifest as tumor-like masses, known as inflammatory pseudotumors, particularly in the hilar region, or as strictures along the bile ducts. These features make IRC an important differential diagnosis for other hepatobiliary diseases, such as primary sclerosing cholangitis (PSC) and cholangiocarcinoma (CCA) [5, 6]. This distinction is challenging because there is no single diagnostic test for a clear diagnosis of IRC. Moreover, IRC is an orphan disease, often leading to limited clinical experience in managing these patients. This often leads to missed diagnoses of IRC in clinical practice, resulting in unnecessary and potentially harmful surgical interventions for suspected malignancy before the condition is confirmed through histopathological analysis [7, 8]. Additionally, many patients suspected of having IRC, often based on elevated IgG4 serum levels, remain without a definitive diagnosis or a clear plan for therapy and surveillance. Therefore, the aim of this article is to provide a practical clinical guide for the diagnosis, differential diagnosis, therapy, and long-term surveillance of patients with IRC.

## Clinical presentation, diagnosis, and differential diagnosis

The typical IRC patient is male (80%–85%), advanced in age (50–75 years or older), and presents with obstructive jaundice, upper abdominal discomfort, and weight loss, raising suspicion of hepatobiliary malignancy [1, 2]. Cholestatic pruritus may be present in some patients, but it is not typical for IRC [9]. Night sweats or fever are more likely to indicate malignancy or infectious complications, such as cholangitis, rather than IRC in adult patients [10]. Due to the frequent association with type 1 AIP, steatorrhea, an indicator of exocrine pancreatic insufficiency—often mild in severity—or pancreatic type 3c diabetes mellitus are possible concomitant conditions [11]. Patients frequently report prolonged occupational exposure (>1 year) to solvents, oil products, dyes, or industrial gases (“blue-collar work”), which is considered a potential risk factor for the development of IRC [12, 13]. This can be regarded as an anamnestic hint for IRC, although the association with disease development remains unclear.

**Figure 1.**
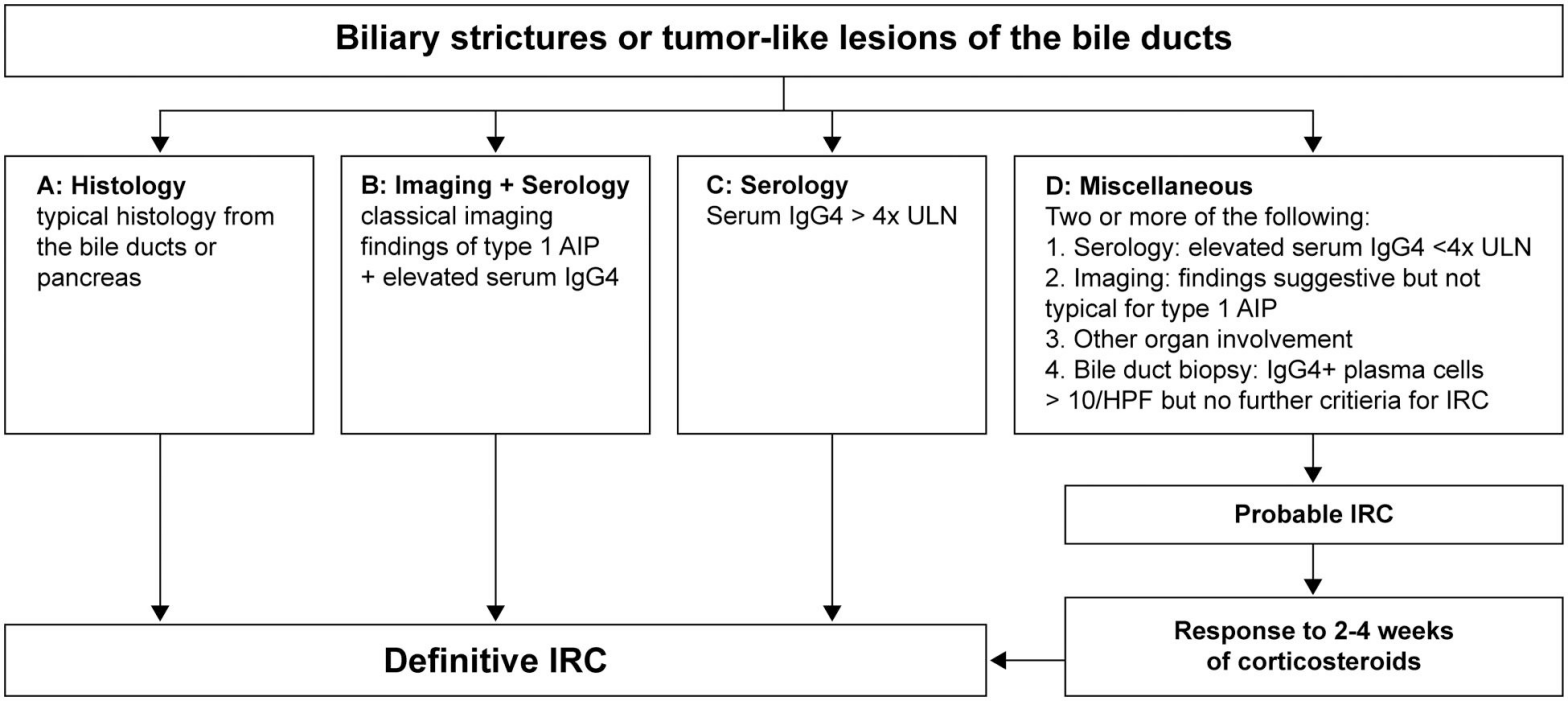
Diagnostic flowchart based on the modified HISORt criteria in suspected IRC. HISORt = histology, imaging, serology, other organ involvement, and response to steroid therapy, HPF = high-power field, IRC = IgG4-related cholangitis, type 1 AIP = autoimmune pancreatitis type 1, ULN = upper limit of normal. Modified from Kersten *et al*. [3].

The diagnosis of IRC is challenging and requires a comprehensive work-up. The necessity of conducting this work-up thoroughly is emphasized by the fact that in up to 10%–15% of patients who underwent major surgery for suspected biliopancreatic malignancy, the histological diagnosis reveals IgG4-RD without any evidence of malignancy. In retrospect, a considerable portion of these cases could have been diagnosed with IgG4-RD based on the available clinical, histological, and imaging data prior to surgery, and the intervention could have been avoided [7, 8]. Multiple diagnostic algorithms have been developed, and the choice of algorithm varies between different medical specialties confronted with the diagnosis of IgG4-RD. While rheumatologists typically follow the 2019 American College of Rheumatology/European League Against Rheumatism Classification Criteria for IgG4-RD [10], the HISORt (histology, imaging, serology, other organ involvement, and response to immunosuppressive therapy) criteria are established as the diagnostic standard for gastroenterologists [14]. HISORt is an acronym for the parameters included in the algorithm: histology (H), imaging (I), serology (S), other organ manifestations of IgG4-RD (O), and response to immunosuppressive therapy (Rt). Figure 1 provides a flowchart based on the modified HISORt criteria for the diagnostic approach to suspected IRC. The individual parameters in the flowchart are explained in detail below.

### Histology (H)

IRC is typically diagnosed histologically, as suspicion often arises from histological findings, though histology is not mandatory. Typical findings include fibrosing-inflammatory lesions with dense lymphoplasmacytic infiltrates of IgG4+ plasma cells, CD4+ T lymphocytes, and eosinophilic granulocytes. Key histopathological features include obliterative phlebitis and storiform fibrosis, a pattern almost exclusive to IgG4-RD [15, 16]. Diagnostic criteria require >10 IgG4+ plasma cells per high-power field (400× magnification) in biopsies or >50 in resection specimens, with an IgG4+/IgG+ ratio > 0.4 [15, 16], as biopsies from patients with PSC or CCA can also contain IgG4+ cells. Due to the uneven distribution of the cells, the high-power field with the highest number is counted.

Endoscopic retrograde cholangiopancreatography (ERCP)-guided bile duct biopsy is the method of choice to obtain a biopsy in patients with suspected IRC. Biopsy accuracy can be improved by prior balloon dilatation [17] or cholangioscopy for better targeting and visualization [18]. In patients with concomitant AIP, a duodenal papillary biopsy can initially be performed [19, 20], followed by an ERCP-guided bile duct biopsy in case of inconclusive results. Liver biopsy is not standard for IRC diagnosis and will not provide diagnostic benefit in the majority of patients [16]. Unlike PSC, isolated small-duct IRC has not been documented.

In any case, histology from lesions that are suspicious for malignancy is necessary, even when the diagnosis of IRC is already established, as CCA can also occur in patients with IRC [21–23].

### Imaging (I)

**Figure 2.**
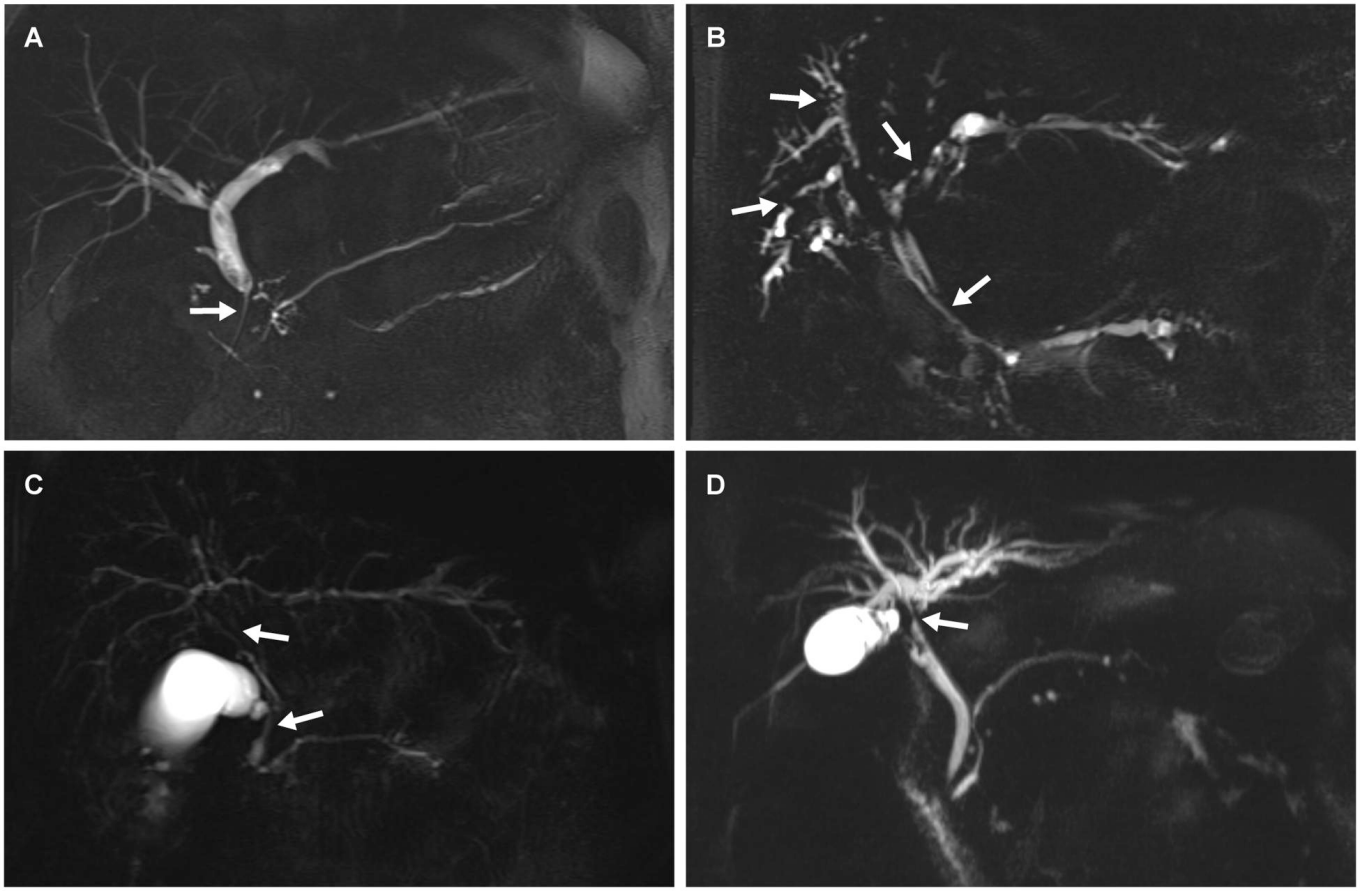
Exemplary MRI/MRCP images of patients with different types of IRC. Affected bile duct areas are marked with arrows. (A) A 52-year-old male patient with an inflammatory stricture of the distal common bile duct (IRC type 1). (B) A 52-year-old male patient with an inflammatory distal stricture of the common bile duct and multiple strictures in the intrahepatic bile ducts with prestenotic dilatations (IRC type 2a). (C) A 59-year-old male patient with a hilar and distal inflammatory stenosis of the common bile duct (IRC type 3). (D) A 45-year-old male patient with an isolated hilar inflammatory stenosis (IRC Type 4). IRC = IgG4-related cholangitis, MRI/MRCP = magnetic resonance imaging/cholangiopancreatography.

The presence of biliary strictures is usually the starting point for considering the possibility of IRC. IRC can involve any segment of the biliary tree and may present as singular or multiple bile duct strictures with wall thickening, or as tumor-like lesions when inflammatory pseudotumors have formed [5, 6]. Depending on the distribution, IRC is classified into four types: type 1 (single distal common bile duct stricture), type 2a/b (distal and intrahepatic strictures with or without prestenotic dilations), type 3 (hilar and distal strictures), and type 4 (isolated hilar stricture), with type 1 being the most common presentation (Figure 2) [24, 25]. Each type is associated with specific differential diagnoses: type 1 resembles pancreatic carcinoma, CCA, or chronic pancreatitis. For type 2, the differential diagnoses for type 1 and PSC must be considered. Type 3 raises suspicion, in addition to the differential diagnoses for type 1, for a Klatskin tumor. Type 4 is highly suggestive for a Klatskin tumor. Imaging should start with a non-invasive modality, such as contrast-enhanced magnetic resonance imaging (MRI)/magnetic resonance cholangiopancreatography (MRCP) and computed tomography (CT). Thickening of the bile duct wall is useful for distinguishing IRC from PSC. In IRC, this thickening typically leads to elongated, band-like constrictions, unlike the shorter, localized strictures seen in PSC. A wall thickness of the common bile duct greater than 2.5 mm on MRI has been suggested as a diagnostic indicator favoring IRC over PSC [6]. The thickening of the bile duct wall does not help in distinguishing between IRC and CCA. However, the onset of strictures can provide valuable information; while IRC generally transitions smoothly into the stricture, an abrupt bile duct cutoff is highly suggestive of CCA [26]. In contrast, the added diagnostic value of 2-deoxy-2-[fluorine-18] fluoro-D-glucose integrated with CT (18F-FDG PET/CT) is limited and not routinely recommended due to radiation exposure and cost [27, 28]. Evidence of extrabiliary involvement, particularly in the pancreas, strongly suggests IRC. Typical imaging findings for type 1 AIP include a diffusely enlarged, sausage-shaped pancreas, edematous fat tissue, and multifocal strictures of the pancreatic duct without upstream dilatation [5].

### Serology (S)

The IgG4 serum level is elevated in 75%–80% of IRC patients (>1.4 g/L) but only confirms IRC in the presence of bile duct strictures when exceeding four times the upper limit of normal (ULN) (>5.6 g/L) [3]. Elevated IgG4 levels are also seen in 10%–15% of PSC, CCA, and pancreatic carcinoma cases, typically <4× ULN [29, 30]. In patients with moderately elevated IgG4 levels (>1.4 to <2.8 g/L), an IgG4/IgG1 ratio > 0.24 favors IRC over PSC [30]. However, histology is necessary in these patients, to confirm the diagnosis of IRC and to exclude CCA. Immediate corticosteroid therapy for IRC without histological confirmation can only be initiated in patients with IgG4 serum levels >5.6 g/L [3, 30]. Carbohydrate antigen 19–9 (CA19–9) is an unreliable serum marker for distinguishing IRC from malignancies, as patients with IRC can also present with significantly elevated CA19-9 serum levels (>1,000 IU/mL) [31]. Emerging biomarkers, such as class-switched IgG4+ B cell receptor clones [32], are not yet clinically available.

### Other organ manifestations of IgG4-RD (O)

IgG4-RD is a systemic disease often with a prolonged, clinically silent course before symptoms arise. In nearly all patients with IRC, at least one other organ is affected either synchronously or metachronously. Commonly involved organs, aside from the previously discussed frequent synchronous occurrence with type 1 AIP, include the salivary glands (5%–26%), kidney (1%–26%), retroperitoneum (3%–17%), lymph nodes (2%–43%), lacrimal glands (8%), lung (1%–7%), eyes (2%–15%), aorta (1%–6%), gallbladder (2%–7%), pleura (5%), hypophysis (2%), prostate (2%), and others [3, 33]. In these organs, asymptomatic courses may occur, sometimes with spontaneous improvement and clinical resolution, mass-forming lesions, or slowly developed organ dysfunction with the typical histological changes of IgG4-RD [33]. Patients should be asked about prior organ involvement, and existing biopsies or imaging should be reviewed for signs of IgG4-RD. However, non-targeted whole-body imaging is not recommended, as findings in other organs are often nonspecific and do not alter the systemic immunosuppressive treatment already required for IRC.

### Response to immunosuppressive therapy (rt)

Response to corticosteroids, the first-line treatment for IRC, is part of the HISORt diagnostic algorithm. An initial dose of 0.5–0.6 mg/kg/day prednisolone (30–40 mg/day for average weight) is recommended. There is a lack of consensus on when this criterion should be considered positive. We advocate that this criterion is positive if, 2–4 weeks after starting treatment, imaging shows a clear improvement in bile duct changes and cholestatic parameters drop below 2× ULN, based on the recommendations of the German and European guidelines [34, 35]. IgG4 serum levels are unlikely to decline significantly within this timeframe due to the 21-day half-life of IgG4 [36].

Corticosteroid response distinguishes IRC from PSC and CCA but should only be initiated for patients meeting criteria for probable or definite IRC (Figure 1). Probable IRC is assumed when bile duct alterations are accompanied by at least two of the following: elevated IgG4 (>1.4 g/L), imaging changes suggestive but not typical of type 1 AIP, other organ involvement, or a bile duct biopsy showing >10 IgG4+ plasma cells without other histological features of IgG4-RD. Definite IRC is diagnosed with bile duct alterations plus one of the following: (A) typical histology, (B) clear imaging criteria of type 1 AIP with elevated IgG4 (>1.4 g/L), or (C) IgG4 > 4× ULN (>5.6 g/L) [3].

If corticosteroids fail, the diagnosis of IRC should be re-evaluated, particularly to rule out CCA. Fibrotic bile duct strictures in chronic IRC may persist despite treatment. In such cases, 18F-FDG PET/CT after 2–4 weeks of corticosteroids may help distinguish active CCA from fibrosis [34]. Repeat biopsies should be performed, and if uncertainty remains, surgical resection must be considered. Table 1 provides an overview of typical findings for distinguishing IRC from PSC and CCA.

**Table 1.** Typical distinguishing features of IRC, PSC, and CCA

Item	IRC	PSC	CCA
Epidemiological characteristic	Male > female, age 50–75 years	Male > female, age < 40 years	Identical to IRC
Histology (H)	Lymphoplasmacytic infiltrate, obliterative phlebitis, storiform fibrosis IgG4+ plasma cells: biopsy > 10/HPF, resection >50/HPF, IgG4+/IgG+ ratio > 0.4	Onion skin fibrosis, fibro-obliterative bile ducts IgG4+/IgG+ typically < 0.4	Dysplasia or malignant cells IgG4+/IgG+ < 0.4
Imaging (I)	Bile duct strictures: long band-shaped strictures; bile duct wall thickening: single wall CBD > 2.5 mm in stricturing area Mass forming: mass in biliary tree and/or liver parenchyma	Bile duct strictures: circumscribed short strictures, beaded biliary tree	Bile duct strictures: short bile duct stricture, bile duct wall thickening
Serology (S)	Serum IgG4+ > ULN 80% [37], > 4× ULN pathognomonic p-ANCA <10% [38] CA19-9 > ULN 30%–50% [31, 39]	Serum IgG4+ > ULN 15%–25% [30, 31] p-ANCA 40% [40] CA19-9 > ULN 12.5% [31]	Serum IgG4+ > ULN 13.5% [29] CA19-9 > ULN 75% [41]
Other organs (O)	Type I AIP > 90%, other organs (often metachronously)	IBD ∼80%	Metastases
Response to therapy (Rt)	Responsive to corticosteroids	Usually not responsive to corticosteroids, except PSC-AIH variant or PSC with high serum IgG4	Usually not responsive to corticosteroids, improvement of inflammatory component in some patients

AIH = autoimmune hepatitis, AIP = autoimmune pancreatitis type 1, p-ANCA = perinuclear anti-neutrophil cytoplasmic antibodies, CA19-9 = carbohydrate antigen 19-9, CBD = common bile duct, CCA = cholangiocellular carcinoma, HPF = high-power field, IBD = inflammatory bowel disease, IRC = IgG4-related cholangitis, PSC = primary sclerosing cholangitis, ULN = upper limit of normal. Modified from Kersten *et al.* [3].

## Therapy

IRC treatment starts immediately upon definite diagnosis, in most cases after confirmation of response to therapy following 2–4 weeks of corticosteroids. The goal of treatment is to suppress inflammation, prevent fibrotic progression, and avert disease-related complications by keeping the disease in a quiescent state [34]. Treatment is structured, as with other autoimmune diseases, into (i) remission induction and (ii) remission maintenance.

### Remission induction

For remission induction, corticosteroids—typically prednisolone—are initially used at a dose of 0.5–0.6 mg/kg/day (30–40 mg/day) [4, 34, 36]. In patients at high risk of steroid-associated side effects (e.g. osteoporosis, diabetes mellitus), a lower prednisolone dose of 10–20 mg/day may be used, which has shown comparable effectiveness for remission induction compared to medium and high doses [42]. However, this recommendation still requires prospective validation and can therefore currently only be made with caution on a case-by-case basis. The starting dose should be maintained for 4 weeks and then reduced by 5 mg every 2 weeks until a maintenance dose of ≤10 mg/day is reached. The maintenance dose is continued for 3–12 months, with the dose and duration adjusted based on the individual course of the disease [4, 34, 36]. Corticosteroids achieve very high response rates, with up to 97% of patients showing partial remission after 3 months of induction therapy with prednisolone, and about two-thirds achieving complete remission (resolution of bile duct strictures/imaging changes and normalization of liver enzyme levels) [2–4]. However, nearly half of the patients experience a relapse after tapering or discontinuing prednisolone, as indicated by a re-increase in liver enzyme levels [4, 34]. These patients require re-induction therapy with increased glucocorticoid dosing, followed by a prolonged taper. To reduce the cumulative dose of corticosteroids and minimize the risk of relapse, adding immunomodulators during the induction phase can be considered. However, it is not clearly established which patients would benefit most, which agent should be used, or the optimal timing for initiation. In patients with previous relapse or high relapse risk (criteria see below), an immunomodulator is highly recommended [34]. There are data available for azathioprine, iguratimod, methotrexate, mycophenolate mofetil, leflunomide, and cyclophosphamide, each showing higher remission rates and/or reduced relapse risk when added to prednisolone, with no current evidence favoring one immunomodulator over another [43–48]. In rare cases of corticosteroid intolerance or non-response, the anti-CD20 antibody rituximab can be considered for remission induction [34, 49, 50]. A recent phase 3, randomized, placebo-controlled trial evaluated inebilizumab, a CD19-targeting B-cell-depleting antibody, in patients with active IgG4-RD [51]. Among 135 participants, inebilizumab significantly reduced the risk of disease flares (10% vs 60% with placebo) and achieved lower annualized flare rates. Additionally, more participants receiving inebilizumab achieved treatment- and glucocorticoid-free complete remission compared to placebo. Thus, inebilizumab may be a promising therapeutic option for IgG4-RD, though further investigation of its safety profile is warranted due to higher serious adverse event rates (e.g. infections, cytopenia)[51]. However, inebilizumab has not yet been specifically tested in patients with IRC and is currently not approved for this indication.

B-cell depleting agents, such as rituximab and inebilizumab, should generally be reserved for exceptional cases. These therapies may significantly increase the risk of infectious complications, such as cholangitis, especially in patients with advanced bile duct strictures and prior interventions [3]. In our clinical practice, remission is typically induced with medium-dose prednisolone for 4 weeks, followed by a stepwise taper to a dose of 5 mg/day. In cases of prior relapse or high relapse risk (criteria see below), azathioprine (starting dose of 50 mg/day) or, alternatively, mycophenolate mofetil is added, generally before beginning the prednisolone taper at week 4 of treatment. In patients without additional immunomodulators, if complete remission is achieved after 3 months of prednisolone alone, we typically end the medication while closely monitoring for relapse. If complete remission has not been reached after 3 months, we then introduce azathioprine or mycophenolate mofetil to reduce the risk of prolonged inflammation, which may not be controlled with low-dose prednisolone alone. Patients treated with additional immunomodulators, or with rituximab, are generally recommended to receive maintenance therapy.

### Remission maintenance

Remission maintenance therapy is necessary for all patients with a previous relapse, high relapse risk, or insufficient response to prednisolone alone, necessitating the addition of an immunomodulator or rituximab for remission induction. Criteria for high relapse risk in IRC are multiorgan involvement of IgG4-RD, high levels of baseline serum IgG4 (>4× ULN), and proximal bile duct stenosis (hilar or intrahepatic localization) [4, 52]. Remission maintenance can, at present, be achieved with four different approaches: (a) low-dose corticosteroids plus an immunomodulator (e.g. prednisolone 2.5–5 mg/day plus azathioprine 50–100 mg/day), (b) low-dose corticosteroids alone (e.g. prednisolone 2.5–5 mg/day), (c) an immunomodulator alone (e.g. azathioprine 50–100 mg/day), or (d) rituximab maintenance (e.g. two 1,000 mg infusions 15 days apart every 6 months) [3, 34]. Low-dose corticosteroids plus an immunomodulator are likely superior to corticosteroids or immunomodulators alone, while rituximab appears to be the most effective in preventing a relapse [47]. However, considering the potential side effects of long-term corticosteroid treatment and the previously discussed cautious recommendation for rituximab in IRC, approach (a) low-dose corticosteroids plus an immunomodulator and (c) immunomodulator alone should be preferred [3, 53]. The optimal duration of maintenance therapy in patients with complete remission is unclear. Based on available data, a duration of 2 years seems reasonable; for patients with multiple previous relapses, ≥3 years, or even indefinite maintenance, may be necessary [3, 54, 55]. The additional use of ursodeoxycholic acid may be considered for its hepato- and cholangio-protective effects [3, 56], as recent insights suggest that impaired cholangiocyte defense contributes to the pathomechanism of IRC [57, 58]. However, since ursodeoxycholic acid treatment has not been studied in IRC, this recommendation is not supported by evidence. Fibrotic bile duct strictures in chronic IRC, which do not adequately respond to immunosuppressive therapy, can be treated endoscopically with balloon dilation or short-term stenting after careful exclusion of malignancy [53].

## Long-term surveillance

In general, lifelong follow-up of patients with IRC is advisable, even in cases of long-term complete remission without relapse or further maintenance therapy [34, 53]; however, the evidence regarding the interval and extent of surveillance, as well as the risks of malignancy and progression, is limited. The aim of follow-up is to (1) detect any relapse, which may also involve organs beyond the bile ducts, (2) monitor for progressive damage to the bile ducts, liver, or, in cases with concomitant type 1 AIP, the pancreas, (3) detect potential malignancy, and (4) in patients on maintenance therapy, identify any therapy-induced side effects [3, 34]. In our clinical practice, patients receiving maintenance therapy are typically seen every 3 months in the outpatient clinic. For patients in stable remission without maintenance therapy, the interval can be extended to every 6 to 12 months. It is important to educate patients on potential clinical signs of a relapse. In addition to the previously discussed symptoms of IRC or type 1 AIP, these may include swelling, inflammatory changes, or unexplained dysfunctions in other organs [33]. Patients may consult other specialists, who should be informed about the IRC diagnosis so that the possibility of IgG4-RD involvement in the affected organ can be considered.

At each outpatient visit of a patient with IRC, liver enzymes and IgG4 serum levels are determined. The objective is to achieve and maintain complete normalization of liver enzymes or, in patients with concomitant liver conditions, a return to baseline levels if known. The role of IgG4 serum levels in assessing remission status, disease activity, or relapse remains unclear. A significant decrease or normalization of IgG4 serum levels is observed in some patients responding to therapy and is even recommended by some authors as a parameter for defining biochemical response [35]. However, in other patients, IgG4 serum levels remain elevated or show no change, despite achieving complete remission with normalization of liver values and resolution of imaging changes [59, 60]. Overall, IgG4 serum levels appear to be unsuitable for monitoring the disease course [3, 34]. Nevertheless, the risk of relapse seems to be higher in patients who do not achieve normalization of serum IgG4 [60]. Thus, in our clinical practice, elevated IgG4 serum levels result in a continued follow-up interval of every 3 months to detect relapse, even in cases of stable remission without maintenance therapy. Due to the close association with type 1 AIP, exocrine and endocrine pancreatic function needs to be monitored [11, 34, 35]. At each outpatient visit, patients should be asked about steatorrhea or weight loss, with fecal elastase measured if these symptoms are present. Glycated hemoglobin and fasting blood glucose levels should be determined annually, in line with the recommendations for patients with chronic pancreatitis [61]. It is unclear whether monitoring for fibrosis progression is necessary in patients with complete remission. However, as in patients with other cholestatic liver diseases [62, 63], annual transient elastography is conducted at our center. It is important that, for patients on maintenance therapy, potential therapy-induced side effects are monitored at every outpatient visit, as detailed in respective guidelines of rheumatological societies [64–66].

There is limited evidence regarding the interval and preferred imaging modalities for follow-up. In patients with IRC-related biliary cirrhosis, semi-annual ultrasound screening for hepatocellular carcinoma is performed [67]. The resolution of imaging changes during induction therapy is assessed 2–4 weeks after the start of treatment and after achieving complete biochemical response, typically after 3 months, using contrast-enhanced MRI/MRCP or CT [34]. Further cross-sectional imaging is probably only necessary if laboratory or clinical evidence of a relapse arises. However, the necessity of follow-up imaging is related to the risk of malignancy, which remains controversial in IRC [9, 11, 55, 68–73]. The risk may be elevated particularly for pancreatic and bile duct cancer within the first year after IRC diagnosis and again more than 5 years after the initial diagnosis [11, 68]. However, a general screening recommendation cannot be made based on the current data. In our clinical practice, annual MRI/MRCP is performed when imaging changes of IRC do not resolve completely. Pancreatic mass-like lesions can be followed up using endoscopic ultrasound. Fibrotic bile duct strictures in chronic IRC may require repeated short-term stenting, which also allows for the identification of new or progressing strictures, as well as for obtaining biopsies from these areas [53]. In patients who achieve complete remission, occasional ultrasound (e.g. every 2–3 years) seems sufficient. When clinical or laboratory indications of a relapse arise, or new-onset weight loss, fever, or night sweats occur, cross-sectional imaging should be performed immediately to detect disease relapse, acute cholangitis, or malignancy.

## Conclusions

In patients with suspected hepatobiliary malignancy, IRC should be considered as a differential diagnosis, especially in cases with inconclusive results. The diagnostic work-up includes a careful anamnesis, MRI/MRCP, IgG4 serology, and histology, preferably obtained from the bile ducts via ERCP-guided biopsy. The HISORt criteria (histology, imaging, serology, other organ involvement, response to therapy) provide a structured approach to diagnosis. Diagnosis is confirmed when bile duct strictures are present alongside (A) typical histology for IRC, (B) typical imaging of concomitant type 1 AIP and elevated IgG4 (>1.4 g/L), or (C) IgG4 serum levels >4× ULN (>5.6 g/L). In patients with a possible but not confirmed IRC (bile duct alterations with elevated IgG4 [>1.4 g/L], imaging changes suggestive but not typical of type 1 AIP, involvement of organs other than the pancreas, or histological detection of IgG4+ plasma cells without clear histologic criteria for IRC), the treatment response to 2–4 weeks of corticosteroids can be assessed, but malignancy must first be ruled out. Treatment of confirmed IRC consists of corticosteroids for remission induction, with gradual tapering over 3–12 months. In patients with a high relapse risk (multiorgan IgG4-RD, IgG4 > 4× ULN, proximal bile duct strictures), maintenance therapy with an additional immunomodulator is recommended. Rituximab may be considered in refractory cases but requires careful risk assessment. Long-term surveillance is necessary to detect relapse, monitor for progressive bile duct, liver, or pancreatic damage, identify malignancy, and assess therapy-induced side effects. Persistent or progressing bile duct strictures require repeated biopsies to exclude malignancy. Structured follow-up is essential, as relapses are frequent and untreated disease may lead to fibrosis, cirrhosis, or secondary complications.

## Authors’ contributions

T.H., M.S., C.E., and F.T. drafted the manuscript. D.G. provided radiological imaging. All authors read and approved the final manuscript.

## Funding 

No funding was received to assist with the preparation of this manuscript.

## Conflicts of interest 

The authors declare that there is no conflict of interests in this work.
